# Treatment of Chronic Alveolar Abscess

**Published:** 1875-08

**Authors:** F. Peabody


### TREATMENT OF CHRONIC ALVEOLAR ABSCESS.

BY F. PEABODY, D. D. S.

Perhaps there is no disease which the dental practitioner is called upon more often to treat, which is more interesting in its character and which is often more perplexing than chronic alveolar abscess. I do not intend in this short paper to enter into a discussion of the pathology of this trouble, or the causes of its obstinate endurance which often combats all our efforts at pacification. Nor of the effect it has upon the general organism and the dental organs in particular. Each operator probably has his favorite method of treatmeut, often attended with success and as often with failures of a most disheartening character.

The object of this paper is to call attention more particularly to a class of fistula of the alveolæ of an indolent character, perhaps as properly denominated sick teeth, and to a method of treatment which has been attended with unexpected and gratifying results. The intelligent and honest dental operator makes it his aim, first, to eradicate the disease, next, to so learn the tooth and its surrounding parts, that there will be no probable chance of its return. In order to do this, after the favorite application has been made to the abscess, the object is to fully and thoroughly fill the root, so that no fiber of nerve shall remain to generate gas and no receptacle shall be left to form a nucleus for the regeneration of the trouble. It is doubtful if many reflect on what action the material used for the root filling may have on the surrounding tissues, the principal object being to obtain something that shall thoroughly fill the space occupied by the nerve, and shall at the same time be indestructible. Cotton, asbestos, gutta percha, gold, os-artificial, flax and silk, each have their defenders aud adherents, and many other articles which have been successful in some cases have been lauded by those who have experienced success in their use.

I am now about to call the attention of the association to a material which I have been using for a little more than two years, and which in no case has refused to fulfill the requirements desired and expected. I doubt if I can better do this than by citing a few cases from my register. I do not claim that the material will always and under all circumstances perform a cure, I merely state what it has done for me.

May, 1873, W. H. D., presented himself with an abscess over the left superior canine tooth, arising from the approximate lateral. This tooth had been filled about two years before and in a few months an abscess had appeared and broken at a point over the apex. I had nearly twelve months before treated this abscess and thought I had cured it, but it soon returned pointing over the canine tooth, I treated it again, but all ordinary means failed. I then cut down over the apex of the lateral, removed the alveolus, and tried with an instrument to break up and tear away the sack, the operation was quite painful and resulted in no good. For eight months I worked on this persistently, using every means in my power to subdue its belligerent aspect. I at length concluded there must be some necrosis of the process or absorption of the root which kept up the irritation and the flow of pus, and determined to extract the tooth, excise the point of the root, polish, fill and replace. The gentleman intended the next day to leave the city for a week or two, requested me to defer the operation until he returned. Wishing to strengthen the root, that it might not break in extracting and as much as possible keep it cool, I filled thoroughly with a piece of lead wire and dismissed him.

On his return at the end of two weeks, I was astonished at finding the fistula absent, the gums perfectly cured and to all appearances in a healthful condition. I concluded not to extract, but to watch the case. In the same mouth was a bicuspid root, second, right superior, on which I wished to set an artificial crown, an abscess on the root had defied all my efforts to control it. On enlarging the canals to receive the gold intended to support the crown, I found each root had perforated the floor of the antrum, and a discharge of pus and sanies followed, on the removal of the instrument, the flow was profuse and no injection was of any benefit. I thought best to remove the root, but by way of experiment filled both roots with lead wire and dismissed the patient for a week. At the end of that time the abscess was gone, the root which was before quite loose was now firm, and the gums were free from inflammation and apparently healthful. I removed a portion of the lead filling and set an artificial crown on root, this root by strain was afterwards split, rendering the crown useless, but up to the present time both lateral incisor and bicuspid root remain free from inflammation, the parts are apparently sound, and no return of the abscess has followed.

Mrs. H. came with a right central incisor which had been in a chronic state of inflammation for over two years. Her dentist had during this time been treating it, had filled the root and tooth three times, and as often removed the fillings on account of pain. No external fistula was present. The crown was a mere shell, had been excavated until nothing but the enamel was left, tooth sore to the touch, gums in an exalted state of inflammation. It was a very doubtful case, did not feel inclined to take it, promised nothing, but being requested to try, treated for a week, subdued the inflammation, filled the root with lead wire, the pulp cavity with oxychloride of zinc and the crown with gold. No trouble has occurred up to the present time. Every time her husband meets me he thanks me for my cure and success in saving this tooth for his wife. I could enumerate many other cases of a like nature, some even worse, presenting no inviting appearance. In no case since I have used the lead filling for the roots, have I failed in curing these indolent cases of chronic fistula. I say cured—for to all appearance they are cured, they may return but some of the worst have stood the test for over two years, which, I trust, is an indication of their future good behavior. What the rationale of this treatment is, I am not prepared to say. Whether it is the perfect filling of the root, particularly the apex, or whether there is some hidden therapeutic property in the lead, I leave for others to say, I merely state facts and results. It is well known that the human system tolerates with more kindness the presence of lead, than any other metal; but as other materials seem to fulfill all the requirements of a perfect root filling, as far as indestructibility and thoroughness are concerned, it seems to me there must be something more than these facts connected with the results produced by lead.

I can find in no authority, any satisfactory explanation of this or any therapeutic property, that lead in its crude metallic state possesses, save its coolness and the possibility that the portion of the metal exposed at the foramen may be slightly decomposed by the fluids that bathe the apex of the root, forming some salt, the astringent property of which may tend to change the action of the disease to one that nature of itself will control. In that case some of the salts of lead would probably produce the same result, those I have not tried and do not know what their effect would be.

In conclusion I will state my method of procedure. Cutting off a strip of common lead from a water pipe, or from sheet lead, I draw it into wire of different sizes. Cutting this into lengths of half an inch, on a glass slab with a spatula, I roll one end to a cone with a sharp point, having ascertained with a few fibres of cotton wound on a broach the size of the foramen, I cut off the sharp point of the cone to about the same size, then, dipping in creosote, the wire is introduced into the canal and carried with force to the apex; unless the foramen is large, the metal being so yeilding, there is little danger of its being carried through, and even in large openings where the end of the cone is regulated to the size of the foramen, with due care, such an occurrence is not liable, piece after piece is introduced and forced up into the canal until all the space occupied by the nerve is filled. The filling thus made is a very perfect one and I find the material easier to handle than any other I know of. Small and tortuous canals are more difficult to fill and require more care.

To some, this method may not be new. To some, the idea may be of benefit. I have written this very hurriedly, at a day's notice and present it to the association for what it may be worth, trusting it may be tried faithfully by those who have

"sick teeth" to contend with, where other remedies have failed, and that they may find their success as gratifying, as I have, in their efforts to overcome this perplexing disease.

